# Mito-nuclear coevolution and phylogenetic artifacts: the case of bivalve mollusks

**DOI:** 10.1038/s41598-022-15076-y

**Published:** 2022-06-30

**Authors:** Alessandro Formaggioni, Federico Plazzi, Marco Passamonti

**Affiliations:** Department of Biological, Geological and Environmental Sciences, University of Bologna, Via Francesco Selmi, 3, 40126 Bologna, Italy

**Keywords:** Molecular evolution, Phylogenetics

## Abstract

Mito-nuclear phylogenetic discordance in Bivalvia is well known. In particular, the monophyly of Amarsipobranchia (Heterodonta + Pteriomorphia), retrieved from mitochondrial markers, contrasts with the monophyly of Heteroconchia (Heterodonta + Palaeoheterodonta), retrieved from nuclear markers. However, since oxidative phosphorylation nuclear markers support the Amarsipobranchia hypothesis instead of the Heteroconchia one, interacting subunits of the mitochondrial complexes ought to share the same phylogenetic signal notwithstanding the genomic source, which is different from the signal obtained from other nuclear markers. This may be a clue of coevolution between nuclear and mitochondrial genes. In this work we inferred the phylogenetic signal from mitochondrial and nuclear oxidative phosphorylation markers exploiting different phylogenetic approaches and added two more datasets for comparison: genes of the glycolytic pathway and genes related to the biogenesis of regulative small noncoding RNAs. All trees inferred from mitochondrial and nuclear subunits of the mitochondrial complexes support the monophyly of Amarsipobranchia, regardless of the phylogenetic pipeline. However, not every single marker agrees with this topology: this is clearly visible in nuclear subunits that do not directly interact with the mitochondrial counterparts. Overall, our data support the hypothesis of a coevolution between nuclear and mitochondrial genes for the oxidative phosphorylation. Moreover, we suggest a relationship between mitochondrial topology and different nucleotide composition between clades, which could be associated to the highly variable gene arrangement in Bivalvia.

## Introduction

### Deep bivalve phylogeny: state-of-art

Bivalves are an extremely diverse group with about 50,000 living species^1^. Deep evolutionary relationships among major clades within the molluscan class Bivalvia are only recently coming to a shared figure. The class is split into two main subgroups, Protobranchia and Autobranchia, whose origins root deep in the middle Ordovician periods^2–6^. Most likely, extant protobranchs resemble the Cambrian forerunners the most, for many molluscan symplesiomorphies are present, like a well-developed foot and true molluscan ctenidia devoted to gas exchange^7,8^; moreover, food is brought to the mouth by palp proboscides. Two sister groups are usually acknowledged within Protobranchia, Nuculida and Solemyida, which are given an ordinal status^2,9–13^; analyses mainly based on molecular markers proposed to exclude the protobranch superfamily Nuculanoidea from Protobranchia and to better place it within Autobranchia^14–17^; the name Opponobranchia was proposed for remaining protobranchs^18^. On the other hand, the clade Protobranchia has been recovered by most of large-scale datasets^19,20^, but with some exceptions^20^. Therefore, the monophyly of this clade still needs to be assessed.

The way of feeding is radically different in Autobranchia (= Autolamellibranchiata *sensu*^18^), whose common ancestor developed a feeding gill, one of the main drivers of the Ordovician bivalve radiation^3^ and led most groups to the key ecological shift towards infaunalization^4,5,21^. Autobranchia is comprised by three major clades (subclasses^22^): Heterodonta (clams, cockles, razor clams, and their kin), Palaeoheterodonta (freshwater mussels and their kin), and Pteriomorphia (mytilids, oysters, scallops, and their kin)^19,23,24^. Moreover, the former subclass Anomalodesmata^22,25–27^ has been found to be nested within Heterodonta^14,15,18,20,28–31^. Currently, Archiheterodonta (order Carditida) are considered sister group to other Euheterodonta, which are further split into Anomalodesmata itself and Imparidentia^15,20,23,26,27,31–33^.

**Figure 1:**
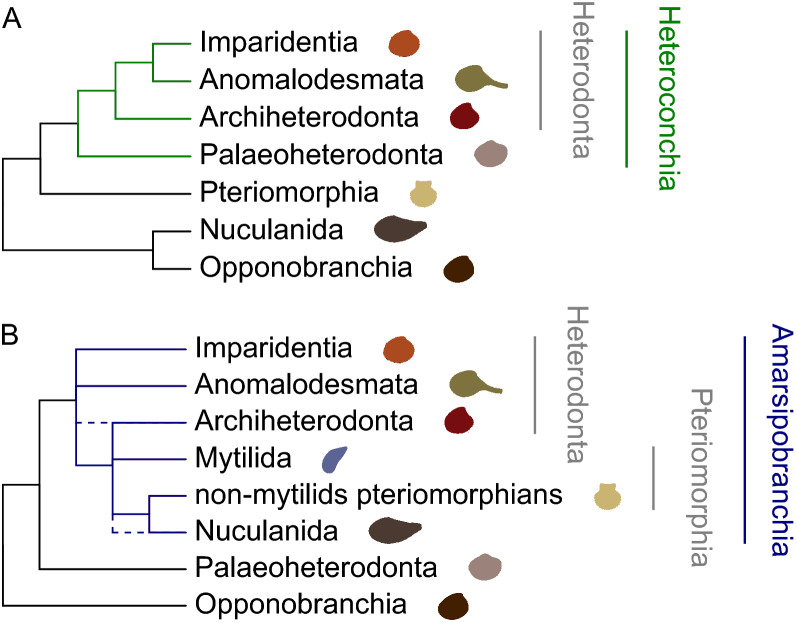
The two main alternative resolutions of the Bivalvia phylogenetic tree. (**A**) The Heteroconchia hypothesis. (**B**) The Amarsipobranchia hypothesis.

Relationships among the main bivalve sub-lineages remained unresolved or uncertain until recently. With minor issues linked to the position of Nuculanida and Anomalodesmata, two main hypotheses have been put forward: the Heteroconchia hypothesis, which involves a sister group relationship between Heterodonta and Palaeoheterodonta (Fig. 1A), and the Amarsipobranchia hypothesis, which involves the sister group relationship between Heterodonta and Pteriomorphia instead (Fig. 1B).

The traditional taxonomic view and morphological analyses of Autobranchia heralded the Heteroconchia hypothesis^4,11,15,26,33,34^; however, a closer relationship between Heterodonta and Pteriomorphia has been suggested following palaeontological evidence^35–40^. The Amarsipobranchia hypothesis was also highly supported by molecular phylogenetics, using mitochondrial markers^15,17,24,41–43^. Contrastingly, the Heteroconchia hypothesis is always supported when nuclear markers are used (either combined with morphological data or not), as well as by means of transcriptomics^15,19,20,33,44–46^. This is a clear example of mito-nuclear phylogenetic discordance^47^.

### The OXPHOS genes and mito-nuclear coevolution

The massive ATP production of aerobic respiration in eukaryotes is mostly made possible through the oxidative phosphorylation (OXPHOS) pathway, which takes place across the inner mitochondrial membrane. OXPHOS pathway is carried out by five enzymatic complexes (CI-V). The genes encoding for the subunits are mostly located in the nuclear genome (around 70 genes), while 13 genes are typically harbored in the mitochondrial genome (mtDNA), at least in most bilaterians. All the complexes but Complex II (CII) have cooperating subunits that are encoded by genes that are located on two different genomes, which show different mutation rate, population size and way of inheritance^48^.

In particular, the low recombination rate of mtDNA leads to the accumulation of slightly deleterious mutations^49^. This process would affect the efficiency of OXPHOS, but slightly negative mutations can be counterbalanced by compensatory mutations in the nuclear genes^50^ or even by new nuclear subunits added to the OXPHOS complexes^51^. According to this model of mito-nuclear coevolution, the process is driven by the accumulation of slightly deleterious mitochondrial mutations, which affects the selective pressure on the interacting nuclear subunits. Indeed, a correlation between the amino acid substitution rate of mitochondrial genes and their interacting nuclear counterparts was shown^52–54^. The evolutionary rate correlation (ERC)^55^ analysis is considered highly reliable to detect signals of mito-nuclear coevolution^56^ and bivalves are among the clades where a positive ERC has been identified^56–58^.

Quite surprisingly, the Amarsipobranchia clade is also supported by nuclear genes encoding for the OXPHOS subunits^57^. Moreover, nuclear and mitochondrial OXPHOS genes show significant ERC and a similar dN/dS ratio^57^ (the ratio between nonsynonymous substitution rate and the synonymous substitution rate^59^).

The mtDNA of bivalves has a highly variable architecture, showing features that are unique among metazoans. Gene order is not conserved inside the class and the high frequency of rearrangements prevents to infer an ancestral gene order for Autobranchia^60^. Among Protobranchia, in the mitochondrial sequence of *Solemya velum* the leading strand, which is also the AC-rich one, harbors the genes *co1, co2, co3, nadh1, nadh2, nadh4, nadh4L and nadh5*, whereas the other strand harbors the genes *atp8, atp6, cytb, nadh1* and *nadh6*^43^. Among Bivalvia, this is likely the most ancestral gene arrangement^43^.

In Palaeoheterodonta the genome organization is highly conserved, and notable rearrangements were never detected within this subclass. Most of the protein coding genes are retained on the GT-rich strand (*atp6, atp8, co1, co2, co3, nadh3, nadh4, nadh4L* and *nadh5*), whereas the other strand harbors *cytb, nadh1, nadh2* and *nadh6*^61^.

Heterodonta and Pteriomorphia show a high degree of rearrangement. Few blocks of genes are shared between different orders of the same clade, and sometimes even among the same family^62^. It is however worth noting that in Heterodonta and Pteriomorphia all genes are retained on one strand, which is rich in G + T. As a matter of fact, the unidirectional replication of the mitochondrial genome leads to an asymmetric nucleotide composition of the two strands, increasing the G + T content in the heavy strand^63^. Most metazoans harbor most of the genes on the light strand, which is rich in A + C, but mollusks show an inverted pattern, in that in these species most of the genes are located on the GT-rich strand^64,65^. The position of mitochondrial genes on different strands has already been reported as a source of phylogenetic artifacts^64,66^. Thus, there could be a relationship between the diverging phylogenetic signal of the mitochondrial markers and the location of some genes in Palaeoheterodonta compared to Heterodonta and Pteriomorphia.

In this study, we performed a phylogenetic analysis using mitochondrial (mt-OXPHOS) and nuclear OXPHOS (nu-OXPHOS) markers, exploiting different phylogenetic approaches. For the sake of comparison, we added two more datasets: genes related to the glycolytic pathway and the genes related to the biogenesis of regulative small noncoding RNAs (sncRNAs). We also analyzed different features of markers selected for phylogenies: how the phylogenetic signal is distributed along the genes, codon usage, amino acid composition and strand location of the markers. We tested possible relationships between these features and the retrieved phylogenetic signals.

Regardless of the phylogenetic method, the Amarsipobranchia are supported only by the OXPHOS markers, both nuclear and mitochondrial. This phylogenetic signal is mostly retained in the organellar markers; among nuclear genes, subunits in direct contact with the mitochondrial counterparts lend most support to this topology. Moreover, we report an unbalanced nucleotide and amino acid composition between Amarsipobranchia and the Palaeoheterodonta, with a higher guanine and thymine content in the latter clade. We suggest that this pattern might be related to a different transcriptional mechanism, which has driven the mitochondrial phylogenetic signal to support Amarsipobranchia.

## Results

### The phylogenetic analysis on the four datasets

The datasets were comprised by 35 species, for four species two mitochondrial haplotypes were sampled (i.e., the female and male mitochondrial haplotypes; see below) (Table 1). All four datasets were incomplete, glycolysis being the most incomplete matrix (Table S1). Conversely, the mt-OXPHOS dataset was the most complete. Species showed a different range of completeness as well: *Myzuhopecten yessoensis* was the most complete species, while the outgroup *Graptacme eborea* was the least complete species (Fig. S1). After the masking step, the mt-OXPHOS dataset was the shortest but also that with the lowest number of discarded sites. The longest dataset was the glycolysis one; the sncRNAs dataset was that with the highest number of discarded sites (Table S1).

**Table 1:** List of species included in the phylogenetic analysis divided by higher classification taxa, orders and families according to Carter and colleagues^118^ and WoRMS database^119^.

Clade	Order	Family	Species
Protobranchia	Nuculida	Nuculanidae	*Ennucula tenuis*
	Solemyida	Solemyidae	*Solemya velum*
	Nuculanida	Sareptidae	*Aequiyoldia eightsii*
Pteriomorphia	Pectinida	Pectinidae	*Amusium pleuronectes*
	Pectinida	Pectinidae	*Mizuhopecten yessoensis*
	Arcida	Arcidae	*Tegillarca granosa*
	Ostreida	Ostreidae	*Magallana angulata*
	Ostreida	Ostreidae	*Saccostrea glomerata*
	Ostreida	Pinnidae	*Pinna atropurpurea*
	Ostreida	Margaritidae	*Pinctada margaritifera*
	Mytilida	Mytilidae	*Bathymodiolus azoricus*
	Mytilida	Mytilidae	*Mytilus edulis* (F and M)
	Mytilida	Mytilidae	*Perna viridis*
Palaeoheterodonta	Unionida	Unionidae	*Cristaria plicata* (F and M)
	Unionida	Unionidae	*Lampsilis cardium*
	Unionida	Unionidae	*Sinohyriopsis cumingii* (F and M)
	Unionida	Maragaritiferidae	*Margaritifera margaritifera*
	Trigoniida	Trigoniidae	*Neotrigonia margaritacea*
Anomalodesmata	Laternulidae	Pandorida	*Laternula elliptica*
	Lyonsiidae	Pandorida	*Lyonsia floridana*
Imparidentia	Venerida	Acticidae	*Arctica islandica*
	Venerida	Cyrenidae	*Corbicula fluminea*
	Venerida	Mactridae	*Mactra chinensis*
	Venerida	Veneridae	*Paratapes textilis*
	Venerida	Veneridae	*Ruditapes philippinarum* (F and M)
	Venerida	Veneridae	*Ruditapes decussatus*
	Venerida	Glossidae	*Glossus humanus*
	Myida	Myidae	*Mya arenaria*
	Sphaeriida	Sphaeriidae	*Sphaerium nucleus*
	Adapendonta	Pharidae	*Sinonovacula constricta*
	Galeommatida	Galeommatidae	*Galeomma turtoni*
Outgroups	Dentaliida	Dentalidae	*Graptacme eborea*
	Octopoda	Octopodidae	*Octopus bimaculoides*
	Chitonida	Acanthochitonidae	*Acanthochitona crinita*
	Lepetellida	Haliotidae	*Haliotis tuberculata*

**Figure 2:**
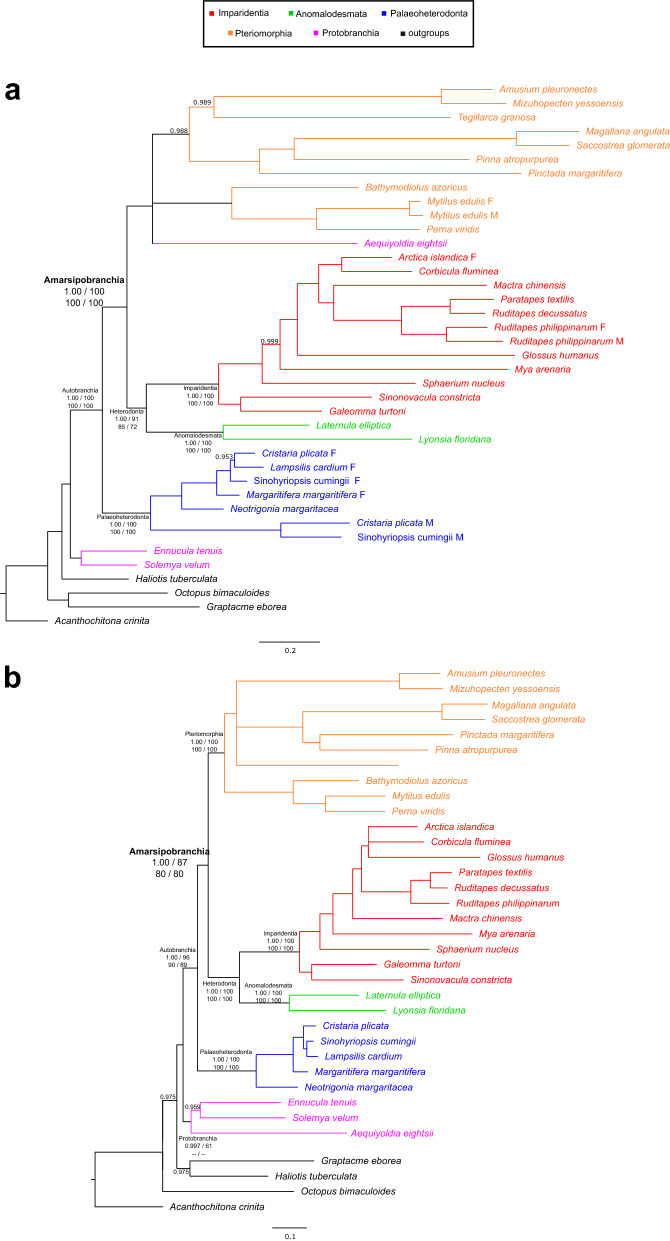
Bayesian trees inferred from the two OXPHOS datasets. (**a**) The mt-OXPHOS tree inferred through MrBayes. (**b**) The nu-OXPHOS tree inferred through MrBayes. Notably, both trees support the Amarsipobranchia hypothesis.The posterior proability on each node is reported when lower than 1.00; nodes with posterior probability lower than 0.95 were collapsed. Major nodes are annotated and support values of each of the four trees inferred for the present work are shown, as follows: MrBayes poterior probability, partitioned and mixture-model IQ-TREE UFBoot values, and RAxML bootstrap value. A double dash instead of the support means that the clade is not monophyletic in that tree. Red, Imparidentia; green, Anomalodesmata; blue, Palaeoheterodonta; orange, Pteriomorphia, purple, Protobranchia; outgroups are shown in black.

The three maximum-likelihood (ML) trees and the single Bayesian tree inferred from the mt-OXPHOS dataset were never significantly different and did not show any alternative resolution of major clades (Fig. 2a, Fig. S2 and Table S2). Protobranchia were basal, exception made for *Aequiyoldia eightsii* (Nuculanida), which clusters within Amarsipobranchia. Autobranchia were fully supported by all four trees. Then, the tree was divided into Amarsipobranchia and Palaeoheterodonta, both fully supported. The Amarsipobranchia were divided into Heterodonta and a clade comprised by *A. eightsii* and Pteriomorphia. Within this clade a polytomy between *A. eightsii,* Mytilida (*Perna viridis*, *Bathymodiolus azoricus*, *Mytilus edulis*) and the other pteriomorphians was recovered. Heterodonta were split into Imparidentia and Anomalodesmata, both fully supported.

The ML and Bayesian trees inferred from the nu-OXPHOS dataset were never significantly different and did not show any alternative resolution of major clades (Fig. 2b, Fig. S3 and Table S2). Protobranchia were basal, but monophyletic in the MrBayes tree only (Fig. 2b); according to the other trees this group was not monophyletic or not robustly supported (Fig. S3). As for the mt-OXPHOS dataset, Autobranchia were split into Palaeoheterodonta and monophyletic Amarsipobranchia. Amarsipobranchia were divided into Pteriomorphia and Heterodonta, and the latter clade was split into Anomalodesmata and Imparidentia; all these clades were fully supported. Within Pteriomorphia, Mytilida are the sister group of remaining OTUs.

**Figure 3:**
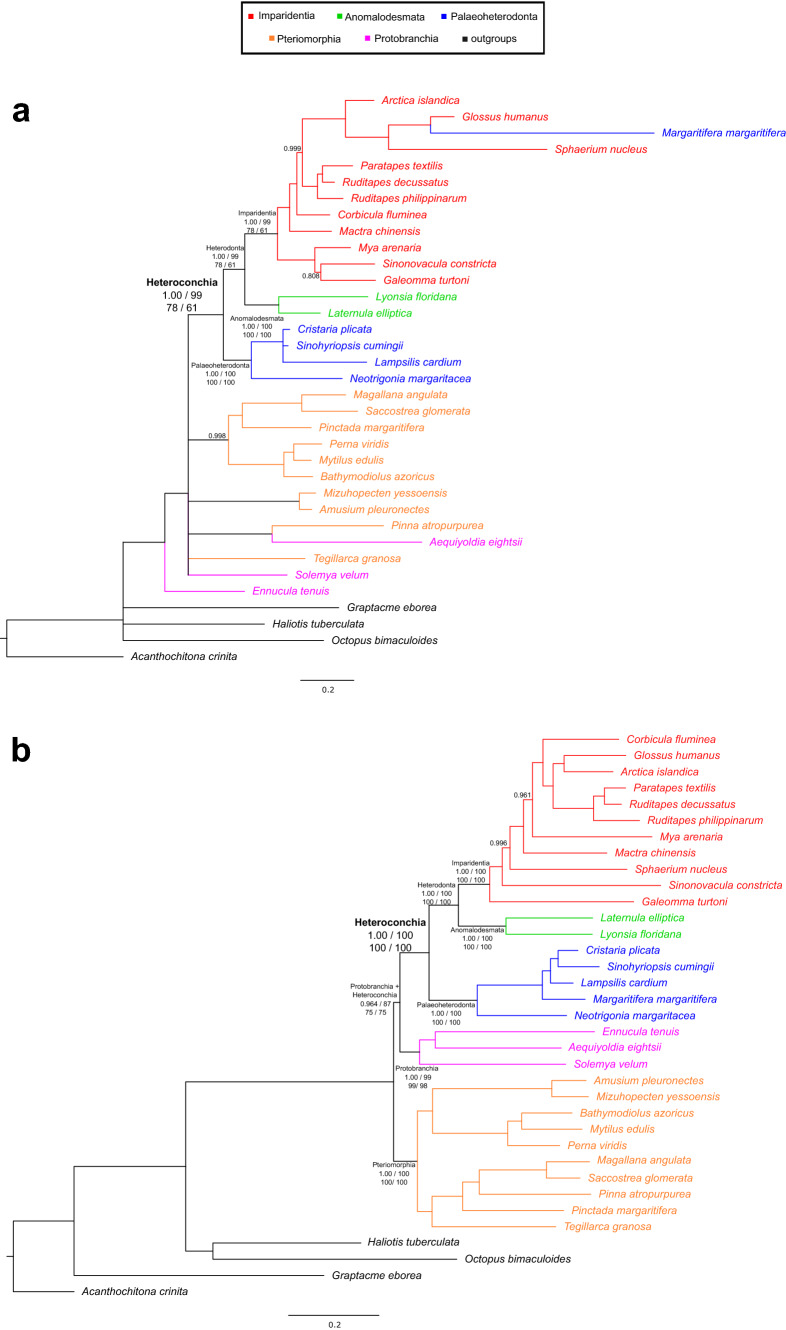
Bayesian trees inferred from the sncRNAs and glycolysis datasets. (**a**) The sncRNAs tree inferred through MrBayes. (**b**) The glycolysis tree inferred through MrBayes. Notably, both trees support the Heteroconchia hypothesis. The posterior proability on each node is reported when lower than 1.00; nodes with posterior probability lower than 0.95 were collapsed. Major nodes are annotated and support values of each of the four trees inferred for the present work are shown, as follows: MrBayes poterior probability, partitioned and mixture-model IQ-TREE UFBoot values, and RAxML bootstrap value. A double dash instead of the support means that the clade is not monophyletic in that tree. Red, Imparidentia; green, Anomalodesmata; blue, Palaeoheterodonta; orange, Pteriomorphia, purple, Protobranchia; outgroups are shown in black.

The ML and Bayesian trees inferred from the sncRNAs dataset were never significantly different and did not show any alternative resolution of the main clades (Fig. 3a, Fig. S4 and Table S2). Overall, several phylogenetic relationships were not resolved and some species were placed in unexpected major clades. After the separation of *Ennucula tenuis*, there was a polytomy with 6 branches: Heteroconchia; Mytilida + Ostreida, exception made for *Pinna atropurpurea*; Pectinida; *A. eightsii* + *P. atropurpurea*; *Tegillarca granosa*; *Solemya velum* (Fig. 3a). Heteroconchia were divided into Palaeoheterodonta and Heterodonta. Heterodonta were split into Anomalodesmata and Imparidentia, even if within the latter clade the palaeoheterodont *Margaritifera margaritifera* was recovered, which does belong to freshwater mussels.

The ML and Bayesian trees inferred from the glycolysis dataset were never significantly different and did not show any alternative resolution of major clades (Fig. 3b, Fig. S5 and Table S2). A long branch led to the Bivalvia node, which further separated Pteriomorphia from other bivalves, leading to the paraphyly of Autobranchia. Namely, Protobranchia and Heteroconchia clustered into a monophyletic group that was supported by all four trees. Heteroconchia were split into Palaeoheterodonta and Heterodonta. The latter clade was divided in Anomalodesmata and Imparidentia; all these clades were fully supported. Within major clades all relationships were resolved and supported and the pteriomorphian and imparidentian species clustered in the expected orders.

Concluding, notwithstanding some issues with the major clade of Protobranchia which blurred the comparison and the substantial overlapping of all phylogenetic trees, the Amarsipobranchia hypothesis was supported in both OXPHOS datasets, while the Heteroconchia hypothesis was supported in the glycolysis and sncRNAs datasets. Henceforth, we will use mt-topology to refer to the Amarsipobranchia hypothesis and nuc-topology for the Heteroconchia hypothesis.

### Phylogenetic signal and its distribution across markers and complexes

Markers belonging to the same dataset may support a different phylogenetic signal. Gene concordance factor (gCF), site concordance factor (sCF)^67^ and ultrafast bootstrap approximation^68^ (UFBoot) were calculated for the Heteroconchia and Amarsipobranchia (which represent alternative resolutions of a node). The mt-OXPHOS dataset showed high support for Amarsipobranchia according to each value (UFBoot = 100; gCF = 30.8; sCF = 48.6), and low support for the Heteroconchia (UFBoot = 0; gCF = 0; sCF = 25.5). Despite a non-zero gCF suggests more markers concordant with the nuc-topology than with the mt-topology, the nu-OXPHOS dataset similarly favors mt-topology (UFBoot = 87; gCF = 3.57; sCF = 37.2) against nuc-topology (UFBoot = 12; gCF = 5.36; sCF = 32.5). Regarding the sncRNAs and glycolysis datasets, markers are more concordant with Heteroconchia, since the UFBoot, gCF and sCF calculated for this topology are considerably higher (Table S3).

For the two OXPHOS datasets we clustered the markers according to the OXPHOS complexes; the sCF for each complex was computed; moreover, it was compared to the sitewise log-likelihood score (SLS) calculated for both topologies. The difference between the mt-topology sitewise log-likelihood score and the nuc-topology sitewise log-likelihood score (ΔSLS) can tell which topology is favored by each site: sites with ΔSLS > 0 supports the mt-topology; sites with ΔSLS < 0 support the nuc-topology. Moreover, by summing all the ΔSLS within a complex we obtained a complexwise log-likelihood score^69,70^ (ΔCLS; Table 2). Since the summed ΔCLS highly depends on the number of sites within each complex, we divided the ΔCLS for the number of sites of each complex (average ΔCLS). For the mitochondrial markers that belong to CI we made a distinction between those *nadh* genes that in Palaeoheterodonta are on the plus strand (CI-ps) from those *nadh* genes located on the minus strand (CI-ms), since we were willing to test if the mt-topology is mostly supported in the genes that are in different strands in Palaeoheterodonta and Amarsipobranchia (i.e. *cytb, nadh1,2,6*; see “Introduction”).

**Table 2:** The phylogenetic signal of nu and mt-OXPHOS markers grouped by complexes.

Group	ΔCLS	Average ΔCLS	%ΔSLSs > 0.5 (%)	%ΔSLSs < − 0.5 (%)	mt-sCF	nuc-sCF
**nu-OXPHOS dataset**						
CV	20.1	0.0079	0.90	0.35	40.9	30.7
CIV	6.2	0.0043	1.39	1.04	42.6	31.0
CIII	8.9	0.0097	1.19	0.59	42.1	33.6
CII	− 7.9	− 0.0073	0.83	0.83	32.9	36.3
CI	− 1.0	− 0.00019	0.96	0.72	35.4	35.0
**mt-OXPHOS dataset**						
CV	5.9	0.0380	3.22	1.29	48.1	26.6
CIV	25.3	0.0272	3.11	0.75	54.6	22.8
CIII (*cytb*)	6.3	0.0181	1.97	0.28	42.6	28.4
CI-ms	11.4	0.0110	2.14	0.77	49.7	24.8
CI-ps	− 7.2	− 0.0090	1.62	0.85	38.1	32.4

The CI mt-markers are split into two groups: CI-ms is comprised by nadh1,2,6 and CI-ps is comprised by nadh3,4,4l,5. For each group it was calculated: ΔCLS; average ΔCLS; percentage of sites with %ΔSLSs > 0.5; percentage of sites with %ΔSLSs < − 0.5; sCF for the mt-topology; sCF for the nuc-topology.

All the mitochondrial groups (Table 2) show a positive ΔCLS; a positive average ΔCLS; more sites that strongly support the mt-topology; more sites in the alignment that agree with the mt-topology. The only exception is CI-ps, where the ΔCLS and average ΔCLS are negative, although the other statistics follow the pattern of the other groups.

Complexes III to V of the nu-OXPHOS dataset (Table 2) support Amarsipobranchia; sites that strongly support the mt-topology (with ΔSLS > 0.5) are more than those supporting the nuc-topology and most sites in the alignment agree with the mt-topology. Contrastingly, CI and CII do not support Amarsipobranchia. In CII there is an equal number of sites for either topology, while in the CI those with a ΔSLS > 0.5 are more. The sCF calculated on the nuc-topology is higher in CII and almost equal in CI with respect to the sCF calculated on the mt-topology.

Overall, in all complexes ΔCLS, average ΔCLS and sCF variate together; statistics related to the strongly supporting sites do not always follow the same pattern, since CI shows a negative ΔCLS but a higher number of sites with ΔSLS > 0.5.

**Figure 4:**
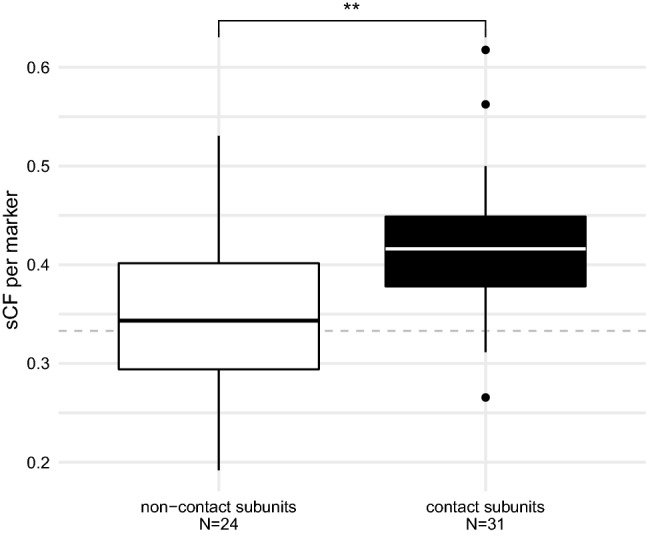
The phylogenetic signal in “contact” and “non-contact” nu-OXPHOS subunits. Boxplot comparing the sCF of nu-OXPHOS markers in direct contact with mitochondrial subunits and the sCF of nu-OXPHOS markers that are not in direct contact. Dashed gray line at 0.33 marks the threshold below which the branch with the highest figure of sCF between the three possible resolutions is not the one that support the mt-topology. Significance calculated through a Student’s t-test (t = − 2.862, *p* value = 0.006363, d.f. = 23, 30).

To test whether the mt-topology phylogenetic signal is mostly retained in the nu-OXPHOS subunits that interact with the mitochondrial subunits, we calculated the sCF referred to each marker and we split the markers into two groups: those that are in direct contact with the mitochondrial counterparts and those that are not. The sCF values of the “contact” nu-OXPHOS markers are significantly higher than the values of “non-contact” nu-OXPHOS makers (*p* value = 0.006363; Fig. 4).

In the sncRNAs and glycolysis datasets the ΔSLS was calculated on the whole matrix. In both datasets the average ΔSLS is negative and there are more sites strongly supporting Heteroconchia (Table S3).

### Nucleotide composition and mitochondrial topology

We placed attention on the nucleotide asymmetry between the two mitochondrial strands, which can be assessed calculating the AT skew and GC skew^71^. In Bivalvia the plus strand is richer in guanines and thymines than the minus strand^64,72^. Thus, we also analyzed possible dissimilarities in the guanine and thymine content (G + T content) between markers that in Pteriomorphia, Imparidentia, Anomalodesmata and Palaeoheterodonta are on the same strand (i.e., *atp6,8, cox1-3, nadh3-5*; Table S4).

**Figure 5:**
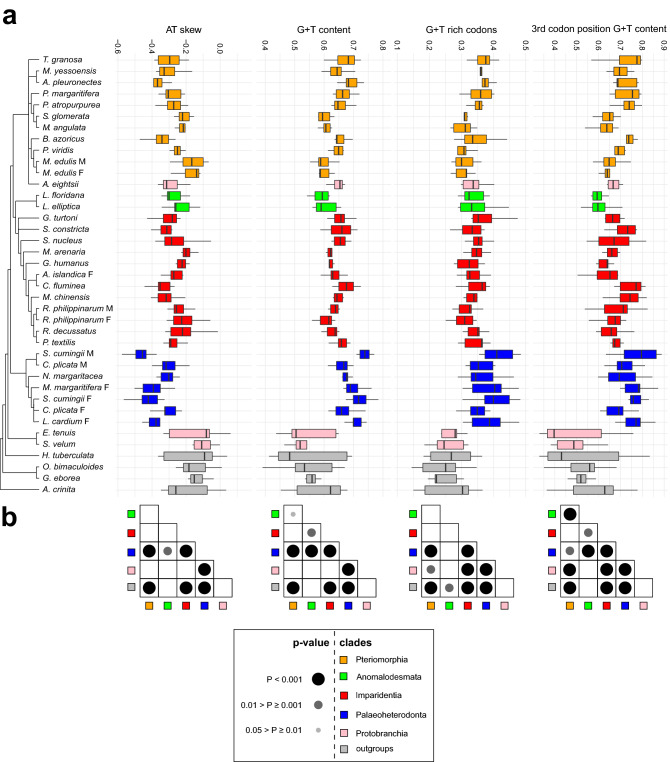
Nucleotide and codon composition statistics in mt-OXPHOS markers compared across OTUs. (**a**) OTUs are reported according to the mitochondrial consensus tree. The x-axis is divided in four boxplots with no outliers, each one reports a different statistic calculated on a set of mt-OXPHOS markers (*atp6,8, cox1-3, nadh3-5*). From left to right, plots report the AT skew, the GT content, the frequency of codons that have T or G at first and second codon position, and the GT content of the third codon position in four-fold degenerated codons, respectively. (**b**) Each table report the significance of pair-wise comparisons between the values reported in the plot right above grouped according to the six clades. The significance is calculated through the Dunn test with the Bonferroni correction. Black and grey dots inside the table mark the significant comparisons; as reported in the legend, the bigger and the darker the dot, the more significant the comparison.

For each mt-OXPHOS marker of each species we calculated the AT skew, the GT content, the frequency of codons with guanines or thymines at the first and the second position (GT-rich codons) and the GT content at the third position of four-fold degenerated codons (Fig. 5). Among Imparidentia, Anomalodesmata, Pteriomorphia and Palaeoheterodonta the markers show an AT skew < 0 and a GT content > 0.5. On average, Palaeoheterodonta show the highest values in all the statistics but the AT skew (Fig. 5a). Indeed, Palaeoheterodonta are always significantly different from the other groups, with the only exception of Anomalodesmata in GT-rich codons (Fig. 5b). On the other hand, the comparisons between Pteriomorphia and Imparidentia are never significant. Regarding the outgroups and Protobranchia values, data show a high standard deviation in most of the cases. The only exception is *A. eightsii,* whose statistics are in line with the values of Imparidentia and Pteriomorphia.

Finally, we studied if the nucleotide compositional patterns outlined in the protein coding regions were extended to the unassigned regions (URs): we downloaded the mitochondrial genomes available on NCBI of all the species that belong to Imparidentia, Anomalodesmata, Palaeoheterodonta, Pteriomorphia and Protobranchia. Then, we calculated the GT content in the URs of the genomes. The GT content of URs calculated on 92 Palaeoheterodonta entries is significantly higher than the one calculated on 77 Pteriomorphia entries, 70 Imparidentia entries and 4 Protobranchia entries. Conversely, it is not significantly higher than the one calculated on 6 Anomalodesmata entries (Fig. S6). For what concerns the other comparisons, no clade is significantly different from any other.

Overall, the nucleotide composition of Palaeoheterodonta mt-OXPHOS markers is most of the times significantly different from the one of other major clades. In particular, we detected a higher GT content. This pattern is reflected in all codon positions as well as in the URs. On the other hand, statistics are overlapping between Imparidentia and Pteriomorphia.

## Discussion

For all four datasets, the more recent nodes were resolved and highly supported. In Imparidentia and Pteriomorphia the OTUs were always placed in the expected orders and major clades (i.e. Pteriomorphia, Anomalodesmata, Imparidentia, Palaeoheterodonta and Protobranchia). Only few exceptions were detected, i.e. the position of *A. eightsii* in the sncRNAs and mt-OXPHOS trees and the position of *M. margaritifera* in the sncRNAs trees. The latter was likely a long branch attraction bias^73^, since the final branches of the OTU and its sister species were the longest in the tree (Fig. 3a). Generally speaking, we regard to these misplacements as minor phylogenetic issues in the broader figure of deep evolutionary relationships among bivalves, which do not significantly blur the topology connecting major clades.

Major clades were retrieved with higher support and with better resolution from the OXPHOS datasets with respect to the glycolysis and sncRNAs dataset. Overall, OXPHOS genes are known to be more conservative, therefore these markers might be more informative in the resolutions of cladogenetic events dating to the Ordovician, approximately 470–480 million years ago (Mya)^36,38,39^.

The mt-OXPHOS trees were mostly coherent with the previous mitochondrial phylogenetic analyses, exception made for the monophyly of the Heterodonta with Anomalodesmata inferred from our analysis^17,24,41,42,57,64,74^. Since all the nu-OXPHOS trees supported the Amarsipobranchia clade (Fig. 1b), our data confirmed that the mt and nu-OXPHOS markers share the same phylogenetic signal, which is different from that inferred from transcriptome-wide analyses or other nuclear markers^14,19,33,44,45^.

Among the interacting sites of coevolving proteins there are epistatic interactions, which lead the sites of both proteins to evolve at the same rate^75^. Bivalvia OXPHOS subunits show a positive ERC, which is the most solid clue of protein coevolution^55,56^. Our data enforce the hypothesis of mito-nuclear coevolution in bivalves, depicting a clear relationship between the phylogenetic signal of interacting subunits. Moreover, they provide an overview on how the phylogenetic signal of OXPHOS subunits may be biased under this type of interaction. The CF and ΔSSL analyses suggested that OXPHOS markers did not equally support the Amarsipobranchia, yet the two dataset were largely coherent with each other: CIII-V markers from both OXPHOS datasets largely supported the mt-topology; contrastingly, CI did not show a clear pattern, and the nuclear-only Complex II favors the nuc-topology. Moreover, the nu-OXPHOS subunits in contact with the mitochondrial counterparts were significantly more concordant with the mt-topology than the subunits that are not directly in contact. Accordingly, previous analyses reported that the CII is the only complex that shows uncorrelated rates of evolution compared to the other subunits^57^.

Finally, the support and concordance statistics (BP, PP, UFBoot, gCF and sCF) calculated for the mt-OXPHOS dataset on the Amarsipobranchia node were always equal to or higher than those calculated for the nu-OXPHOS dataset. Thus, the mt-topology in the first dataset was more consistent: more sites and markers agreed with this topology and the signal was less susceptible to resampling.

The mito-nuclear coevolution is expected to be mainly driven by slightly deleterious mitochondrial mutations that are compensated by the nuclear genome^48,50^. Even if previous data did not show signal of nuclear compensation^57^, it is tempting to conclude that the mitochondrial genome acquired the mutations leading to the mt-topology at first, and then the phylogenetic signal has been traced by interacting sites in the nuclear markers through nuclear compensation.

Pteriomorphia and Imparidentia share some unique mitochondrial features: their gene order is highly rearranged, but all genes are on the same strand. Contrastingly, Palaeoheterodonta show a highly conserved gene order, with a set of genes on the minus strand (*nadh1,2,6* and *cytb*; see Introduction for further details). According to the nucleotide composition analyses, the Palaeoheterodonta mt-OXPHOS markers were significantly GT-richer in each codon position as well as in URs, while Pteriomorphia and Imparidentia did not show any significant difference (Fig. 5, Fig. S6). Thus, this pattern was accounted for either synonymous and non-synonymous substitutions and it was extended also to URs. Mito-nuclear coevolution largely explains why the nu and mt-OXPHOS markers support a common topology.

Mito-nuclear discordance is a quite common phenomenon and a multitude of processes can cause it^76^. The introgression of mitochondrial lines from a phylogenetically distant population is widely used to explain mito-nuclear discordance^47,76^. In some cases it has been hypothesized that a set of nuclear genes might cointrogress to avoid mito-nuclear incompatibilities^77^. The mito-nuclear cointrogression would explain very well our data, since the phylogenetic artifact is mostly supported by the nuc-OXPHOS markers that directly interact with the mt-OXPHOS markers, whereas almost all mt-OXPHOS markers support the mt-topology. In this case, the use of a single mitochondrial strand and other features would be apomorphies arisen along a single branch and subsequently acquired by the other branch through introgression. Having said that, other evidences of cointrogression are limited and only restricted to populations within the same genus^77–79^. In our case the discordance mainly resides in the resolution of deep nodes, which originated around 480 Mya^5,36,80^, between clades that already evolved quite different life habits^5^. Under this scenario, the mito-nuclear cointrogression might not be the most likely hypothesis.

Another source of mito-nuclear discordance can be found in how markers are located on the two mitochondrial strands^66^. In mollusks, whose mitochondrial genome is highly rearranged, the nucleotide bias is also reflected in amino acid bias^64^. Our results showed that the signal supporting the mt-topology (the Amarsipobranchia clade) is not only retained in the set of markers that in Palaeoheterodonta are on the minus strand. Instead, the mt-OXPHOS CIV-V markers on the plus strand clearly favor the Amarsipobranchia hypothesis (Table 2). The higher GT content in Palaeoheterodonta is consistent throughout different parts of the mitochondrial genome, from coding to unassigned regions. Therefore, the nucleotide substitutions that have led to this pattern are likely to be produced by a process that act on the whole genome. Possible candidates might be the mitochondrial transcription and replication, which are indeed notable source of deamination^63,81^. Moreover, mitochondrial replication constitutes the main source of mitochondrial point mutations, at least in humans^82^.

The position of all genes on the same strand is probably linked to the fact that the two clades do not show any significant difference in GT content. It is tempting to hypothesize that transcription involves the coding strand only for these mtDNAs and, thus, the aforementioned deamination effect may be less pronounced. Indeed, even those sncRNAs that were recently described in the imparidentian *R. philippinarum* were annotated on the same coding strand^83^, thus corroborating the idea that only one strand is transcribed in these clades.

The use of a single strand seems also linked to the mitochondrial architecture: among most of the metazoan taxon that share this feature it has been detected a higher mitochondrial rearrangement rate^24,84,85^. Likewise, Pteriomorphia and Imparidentia show highly rearranged mitochondrial genomes^62^. An additional clue is the behavior of *A. eigthsii*: the protobranch species cluster with Pteriomorphia and shows similar nucleotide composition features (Fig. 2a, Fig. 5a). Indeed, although no mitochondrial genome has been annotated from the order Nuculanida, it is possible that *A. eigthsii* mitogenome harbors all the genes on the heavy strand, since all its mitochondrial genes show AT skew < 0 and GC skew > 0 (Table S5).

If the hypothesis holds true, it is reasonable to consider the different transcriptional patterns among Bivalvia as the most likely source that has led the mitochondrial genome to support a different phylogenetic signal, namely a biased one. Since we demonstrated that also non-synonymous mutations have shaped the GT content pattern, the modifications of the amino acid sequences could have altered the epistatic interactions between nuclear and mitochondrial OXPHOS subunits, leading the OXPHOS markers to support the same phylogenetic artifact.

## Conclusions

The results obtained from the phylogenetic analysis of Piccinini and colleagues^57^ has been confirmed by our work, since markers of both OXPHOS datasets support the same biased topology, regardless of the phylogenetic pipeline used. Moreover, we depicted how the coevolution process affected the phylogenetic signal in different set of OXPHOS markers, concluding that the artifactual topology is mainly supported by the OXPHOS subunits that interact more directly.

Considering that the phylogenetic signal is more stable and stronger in the mt-OXPHOS markers, we suggest that the biased topology arose for these markers at first, then it has been acquired also by the nu-OXPHOS markers through the coevolution of interacting subunits. This model agrees with the pattern of evolution hypothesized for the mito-nuclear coevolution. That is, the mito-nuclear coevolution is mainly driven by slightly deleterious mitochondrial mutations that are compensated by the nuclear genome^48,50^.

Our data suggest a relationship between the mt-topology supporting Amarsipobranchia and the gene rearrangements in the Bivalvia mitochondrial genome. The clades that harbor all the mitochondrial genes on a single strand and show a similar nucleotide composition (Pteriomorphia, Heterodonta, and possibly *A. eightsii*) are grouped together in a monophyletic clade. On the other side, Palaeoheterodonta show a peculiar nucleotide composition, which is not only due to the genes located on the minus strand. Indeed, genes such *cox1-3*, *atp6,8, nadh3-5*, even if they are located on the plus strand, show a higher GT content compared to the Amarsipobranchia ones. Overall, the difference in GT content between OTUs may be a source of possible phylogenetic artifacts. Further analyses will be focused on understanding how the nuclear subunits compensated differently during the evolution of Palaeoheterodonta, Pteriomorphia and Heterodonta.

Finally, according to the data, the reliability of the Amarsipobranchia clade should be reconsidered. At the state of the art, although many mitochondrial phylogenies confirmed the Amarsipobranchia clade^24,57,64,74^, no phylogeny supports Amaripobranchia when based on nuclear markers (exception made for the nu-OXPHOS markers^57^; Fig. 2b, Fig. S2). On the other side, the Heteroconchia clade has been retrieved by genome-wide, transcriptomic, and morphological analyses^19,33,44,45^. If the evolutionary scenario depicted in our discussion is correct, then the taxon Amarsipobranchia cannot be supported anymore and has to be considered a phylogenetic artifact: the Heteroconchia clade should be regarded as a more reliable hypothesis instead.

## Materials and methods

### The datasets

Our phylogenetic analyses were performed on four datasets: mt and nu-OXPHOS genes, glycolytic pathway genes, and genes related to the biogenesis of sncRNAs. All markers were retrieved from the transcriptomes used by Piccinini and colleagues^57^: the transcriptomes of 35 molluscan species were assembled (Table 1). When available, the mt-OXPHOS markers from both sexes were retrieved for those species that show mitochondrial Doubly Uniparental Inheritance (DUI^86–88^).

Information about the assembly of transcriptomes is detailed in the aforementioned paper^57^. Briefly, the annotation of transcripts was performed using BLASTx^89^ against a user-defined database and HMMER^90^ against the Pfam database 30.0^91^; the user-defined database contains sequences of all genes of that dataset available for Bivalvia on NCBI.

Clam homologs for the first three datasets were extracted following the gene lists available in the Kyoto Encyclopedia of Genes and Genomes (KEGG^92–95^; https://www.kegg.jp), which provides a curated database of enzymes involved in specific biochemical pathways: namely, the Oxidative phosphorylation pathway (KEGG entry: map00190) and the Glycolysis/Gluconeogenesis pathway (KEGG entry: map00010). Regarding genes for the fourth dataset, i.e. genes related to the biogenesis of sncRNAs, we identified a set of genes shared across Metazoa^96,97^. Entries available on NCBI and UniProt^98^ were included in the database (Table S6). Annotation was performed using BLASTp^89^.

Paralogs were recurrent among the markers associated to glycolysis. Therefore, we devised a method to conservatively distinguish paralogs from orthologs. We inferred the ML tree from each single marker putting orthologs together, which was obtained using IQ-TREE1.7^99^ with mixture model as model of evolution, 1000 UFBoot^68^ replicates, and constraining the Bivalvia clade. Through the analysis of topologies, more than one group of clear monophyletic orthologs were detected in some cases, namely in the markers with KEGG ID K00002, K00128, K00129, K00149, K00627, K00844, K01596, K01623, K01689, K01785, K01895, K03103, K08074, and K13953 (Table S7). In these cases, groups of orthologs were split and considered as single markers. Aiming to ensure that the phylogenetic signal supported by the glycolysis matrix after this scrutiny was coherent, we retained two different datasets associated to glycolysis genes: a larger dataset with all markers obtained in this way (total-glyco) and a dataset with markers that showed no evidence of paralogs (partial-glyco). All subsequent analyses were carried out independently for both datasets; since differences in results were negligible, we are confident that we identified paralogs correctly, thus in the results we mean the total-glyco dataset only when referring to the “glycolysis” dataset.

### Phylogenetic reconstruction

We performed the phylogenetic analysis using amino acid sequences, since we were more interested in deep relationships and nucleic sequences are bound to saturate along long branches. First, we aligned sets of homologous markers with PSI-Coffee^100^. Then, to remove the uninformative or misleading sites for the analysis, we used and combined the results of five different masking algorithms^24^: BMGE^101^, Aliscore^102^, Gblocks^103^, ZORRO^104^ and Noisy^105^. This step was performed by masking_package 1.1, downloaded from GitHub and available at https://github.com/mozoo/masking_package. To include the indels in the phylogenetic reconstruction we ran GapCoder^106^ on every alignment.

To assign the best-fitting evolutionary model to each marker of the matrix we used PartitionFinderProtein^107^. All markers belonging to the same dataset were concatenated together. For each dataset we obtained four trees. (1) One tree was obtained through IQ-TREE 1.7 with the dataset partitioned according to the PartitionFinder results. (2) One tree was obtained through IQ-TREE 1.7 with the mixture model as model of evolution^99^. (3) One tree was obtained through RAxML version 8.2.11^108^ with the dataset partitioned according to the PartitionFinder results, using the CAT model instead of the Gamma model^109^. 1000 bootstrap replicates were executed for each run, to test the robustness of the nodes, and the UFBoot approximation was chosen for IQ-TREE. (4) The fourth tree is based on the Bayesian inference, obtained through MrBayes^110^ with the dataset partitioned according to the PartitionFinder results. Number of generations was set to 10,000,000; the convergence between runs were manually checked to set the burn-in value. To set this value, we looked at the standard deviation of average split frequency over generations; moreover, we took the Potential Scale Reduction Factor (PSRF^111^) into consideration. In each analysis the monophyly of Bivalvia was constrained and in the Bayesian analysis the outgroup was set to be the polyplacophoran *Acanthochitona crinita* (Table 1).

### Analyses on topologies and markers

At the end of phylogenetic analysis, four trees were obtained for each dataset through four different pipelines, as described above. To test whether the trees obtained from the same dataset are significantly different or not, we performed the Shimodaira-Hasegawa test (SH-test^112^), exploiting the RAxML option “-f H”.

The support of each site for the Amarsipobranchia hypothesis (“mt-topology”) and the Heteroconchia hypothesis (“nuc-topology”) was calculated through the ΔSLS. Sites with ΔSLS > 0.5 or ΔSLS < − 0.5 were retained as sites with strong support for either hypothesis^69,70^. To calculate the sitewise log-likelihood we exploited the RAxML option “-f g” providing the RAxML ML tree when the sitewise log-likelihood was calculated on the mt-topology. A tree with the nuc-topology was obtained by running the phylogenetic analysis with the same settings, but constraining the Heteroconchia clade (as suggested by Shen and colleagues^70^), and the resulting ML tree was used to calculate the nuc-topology sitewise log-likelihood.

The sCF and the gCF were calculated through IQ-TREE 1.7 (again with 1000 UF-bootstrap replications) with the option “–cf-verbose” to study phylogenetic signal between and within partitions^67^. Each dataset was partitioned into single markers in order to calculate the sCF per marker and the gCF. Then, the matrices were partitioned according to complexes to obtain sCF per complex. The nu-OXPHOS subunits in direct contact with the mitochondrial counterparts were defined according to the list of Piccinini and colleagues^57^.

Custom-tailored python and R^113^ scripts were used to analyze and plot the nucleotide and amino acid composition, using Biopython^114^ and ggplot2. Since mitochondrial URs are missing from transcriptomes, their nucleotide composition was calculated for a list of NCBI indexes obtained through the alMighto database^65^: a single entry was selected for each species in the database belonging to Palaeoheterodonta, Imparidentia, Anomalodesmata, Pteriomorphia or Protobranchia.

For DUI species the mtDNA of both sexes was selected. The guanine and thymine content in URs was obtained through a customized version of the HERMES tool^115^. The significance of the comparisons was calculated through the Kruskal and Wallis test^116^, followed by the Dunn’s test^117^ with Bonferroni’s correction.

## Supplementary Information

Supplementary Information.

## Data Availability

The data underlying this article are available in the GenBank Nucleotide Database at https://www.ncbi.nlm.nih.gov/ and in the SwissProt database at https://www.uniprot.org/, and can be accessed with the accession numbers provided in the article and in the supplementary materials.
