# Supplementary material for: Mito-nuclear coevolution and phylogenetic artifacts: the case of bivalve mollusks
Source: Sci Rep. 2022 Jun 30;12:11040. doi: 10.1038/s41598-022-15076-y (PMC9247169; doi:10.1038/s41598-022-15076-y)
Supplement: Supplementary file 1 — Supplementary Information. [file 41598_2022_15076_MOESM1_ESM.pdf]

Supplementary materials

Dataset	Matrix completeness	Length masked datasets (aa)	Discarded sites
mt-OXPHOS	95.46%	2,760	26.56%
nu-OXPHOS	72.36%	11,760	37.35%
sncRNAs	88.71%	3,944	72.71%
glycolysis	54.00%	15,852	40.93%

Table S1. Completeness and length of the four datasets. The completeness is the number of sequences of each dataset over the number of hypothetical sequences if each marker would have had a sequence for every OUT. The percentage of discarded sites refers to the sites removed during the masking step.

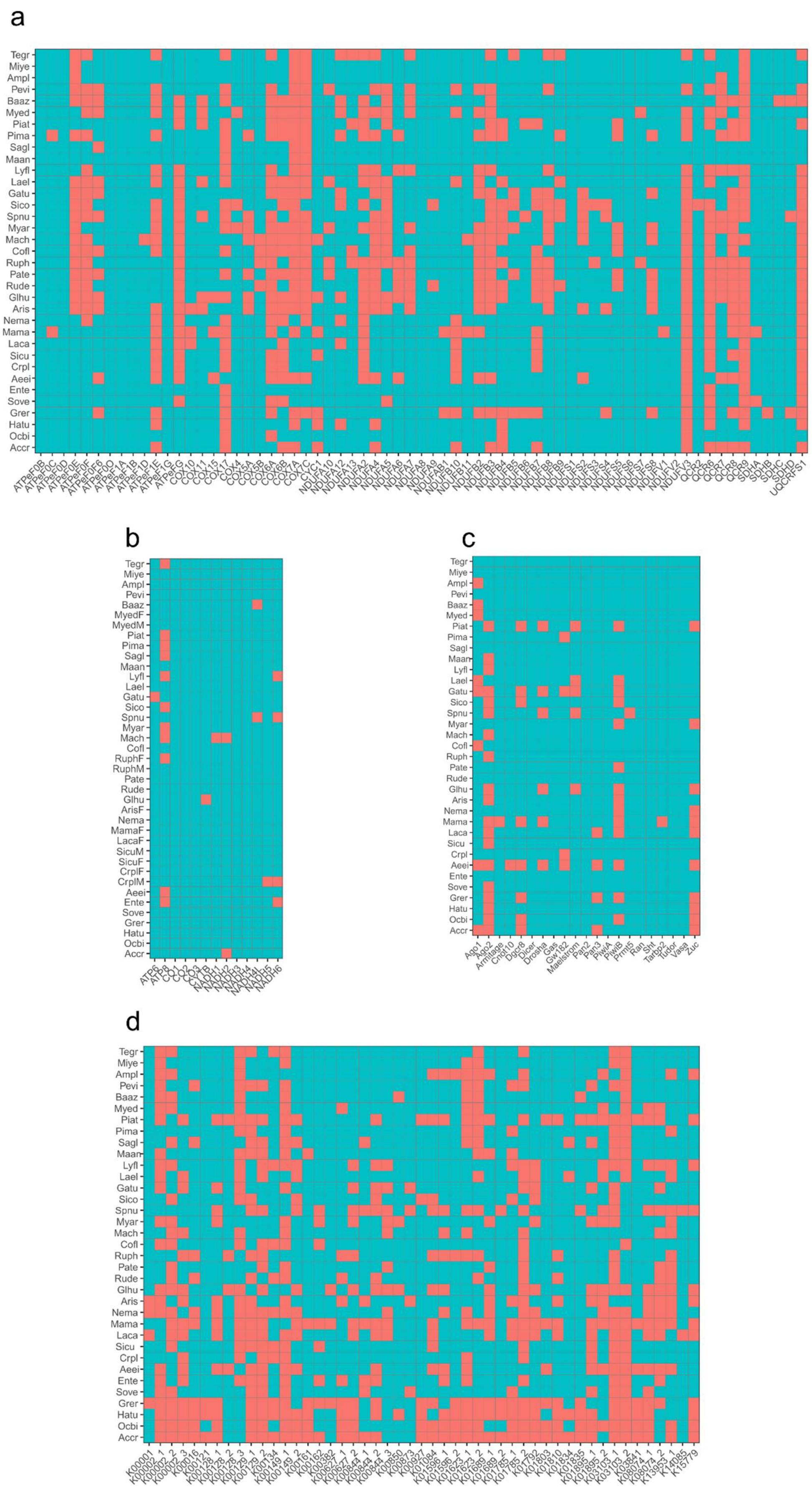
a
Tegr
Miye
Ampl
Pevi
Baaz
Myed
Piat
Pima
Sagl
Maan
Lyfl
Lael
Gatu
Sico
Spnu
Myar
Mach
Cofl
Ruph
Pate
Rude
Glhu
Aris
Nema
Mama
Laca
Sicu
Crpl
Aeei
Ente
Sove
Grer
Hatu
Ocbi
Accr
b
Tegr
Miye
Ampl
Pevi
Baaz
MyedF
MyedM
Piat
Pima
Sagl
Maan
Lyfl
Lael
Gatu
Sico
Spnu
Myar
Mach
Cofl
RuphF
RuphM
Pate
Rude
Glhu
ArisF
Nema
MamaF
LacaF
SicuM
SicuF
CrplF
CrplM
Aeei
Ente
Sove
Grer
Hatu
Ocbi
Accr
ATP6
ATP8
CO1
CO2
CO3
CYTB
NADH1
NADH2
NADH3
NADH4
NADH4L
NADH5
NADH6
c
Ago1
Ago2
Armitage
Cnot10
Dgcr8
Dicer
Drosha
Gas
Gw182
Maelstrom
Pan2
Pan3
PiwiA
PiwiB
Prmt5
Ran
Sht
Tudor
Vasa
Zuc
d

Figure S1. Presence and absence table of each marker in each OTU. On the y-axis the OTUs are shown: each label is abbreviated using the first two letters of the generic name and the first two letters of the specific epithet; F and M are used to distinguish female-type and male-type haplotypes. On the x-axis the markers used in the phylogenetic analysis are shown. Squares are colored in light blue, when the marker is present, or in red, when the marker is absent. (A) The nuclear OXPHOS markers' presence-absence table; (B) the mitochondrial OXPHOS markers' presence-absence table; (C) the sncRNA markers' presence-absence table; (D) the glycolysis markers' presence-absence table (see Table S7).

Dataset	Pipeline	best tree lnL	ΔlnL	SD	P-value
mitochondrial OXPHOS	RAxML	−124,547.30695			
	IQ-TREE partitioned		−3.36015	11.17877	> 0.05
	IQ-TREE mixture		−10.40756	15.82649	> 0.05
	MrBayes		−10.35857	15.85253	> 0.05
nuclear OXPHOS	RAxML		−0.680621	16.37446	> 0.05
	IQ-TREE partitioned		−2.072105	17.540151	> 0.05
	IQ-TREE mixture	−303,787.7888			
	MrBayes		−2.944056	24.151885	> 0.05
sncRNA	RAxML	−98,413.79378			
	IQ-TREE partitioned		−11.724517	43.570374	> 0.05
	IQ-TREE mixture		−9.772834	45.200477	> 0.05
	MrBayes		−1.902815	12.893494	> 0.05
glycolysis	RAxML		−0.000002	0.003007	> 0.05
	IQ-TREE partitioned		−0.000001	0.003283	> 0.05
	IQ-TREE mixture		−0.000001	0.003283	> 0.05
	MrBayes	−282,343.47273			

Table S2. Results of the Shimodaira-Hasegawa test computed between trees inferred from the same dataset but with different phylogenetic pipelines. For each dataset it is reported the log-likelihood of the maximum-likelihood tree (best tree lnL); the difference between the log-likelihood of each tree and the maximum-likelihood tree (ΔlnL); the standard deviation (SD); the P-value.

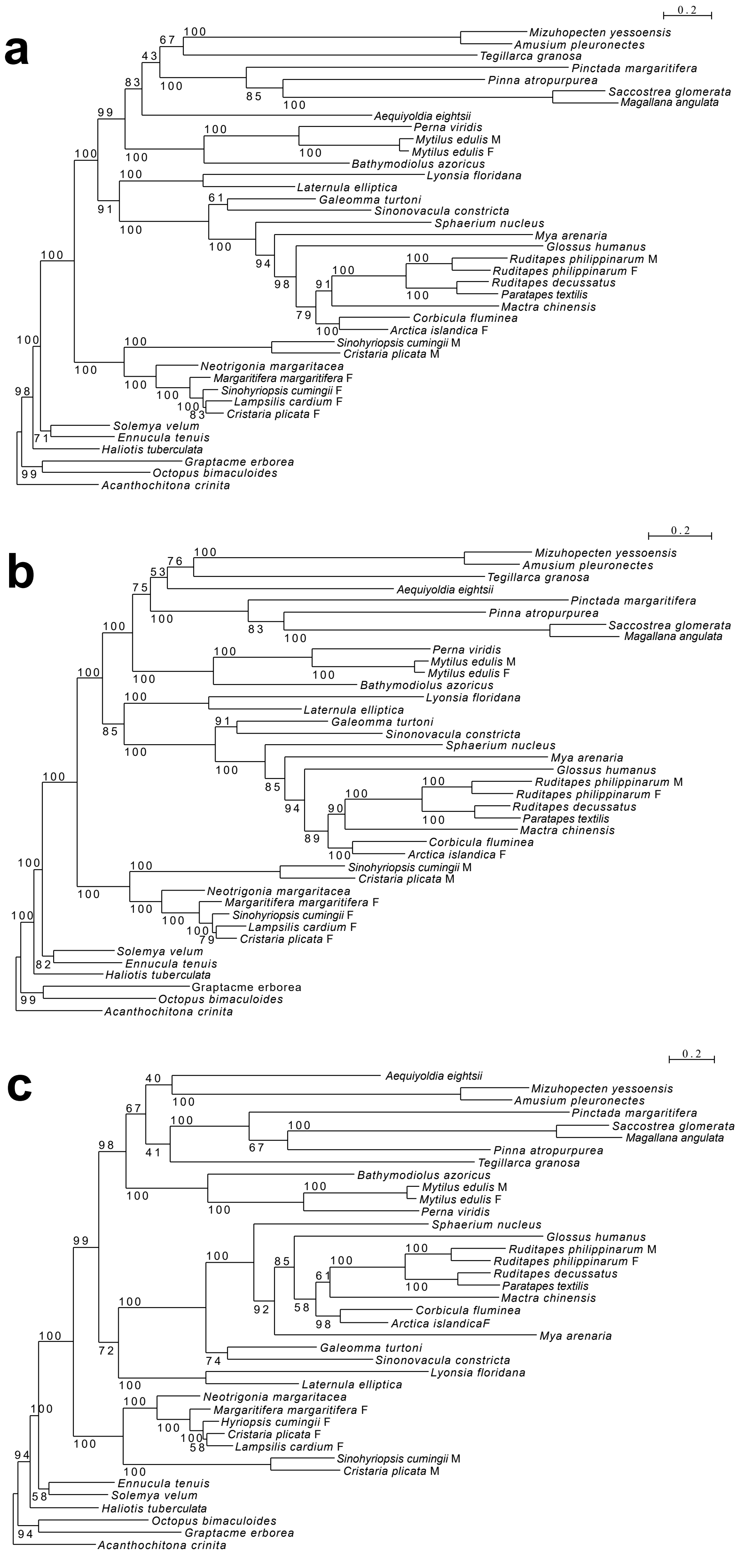

Figure S2. Trees inferred from the mitochondrial OXPHOS dataset. Each tree was obtained through a different phylogenetic pipeline (A) IQTREE partitioning the dataset, node support expressed as UFBoot value; (B) IQTREE with mixture model, node support expressed as UFBoot value; (C) RAxML, node support expressed as bootstrap value.

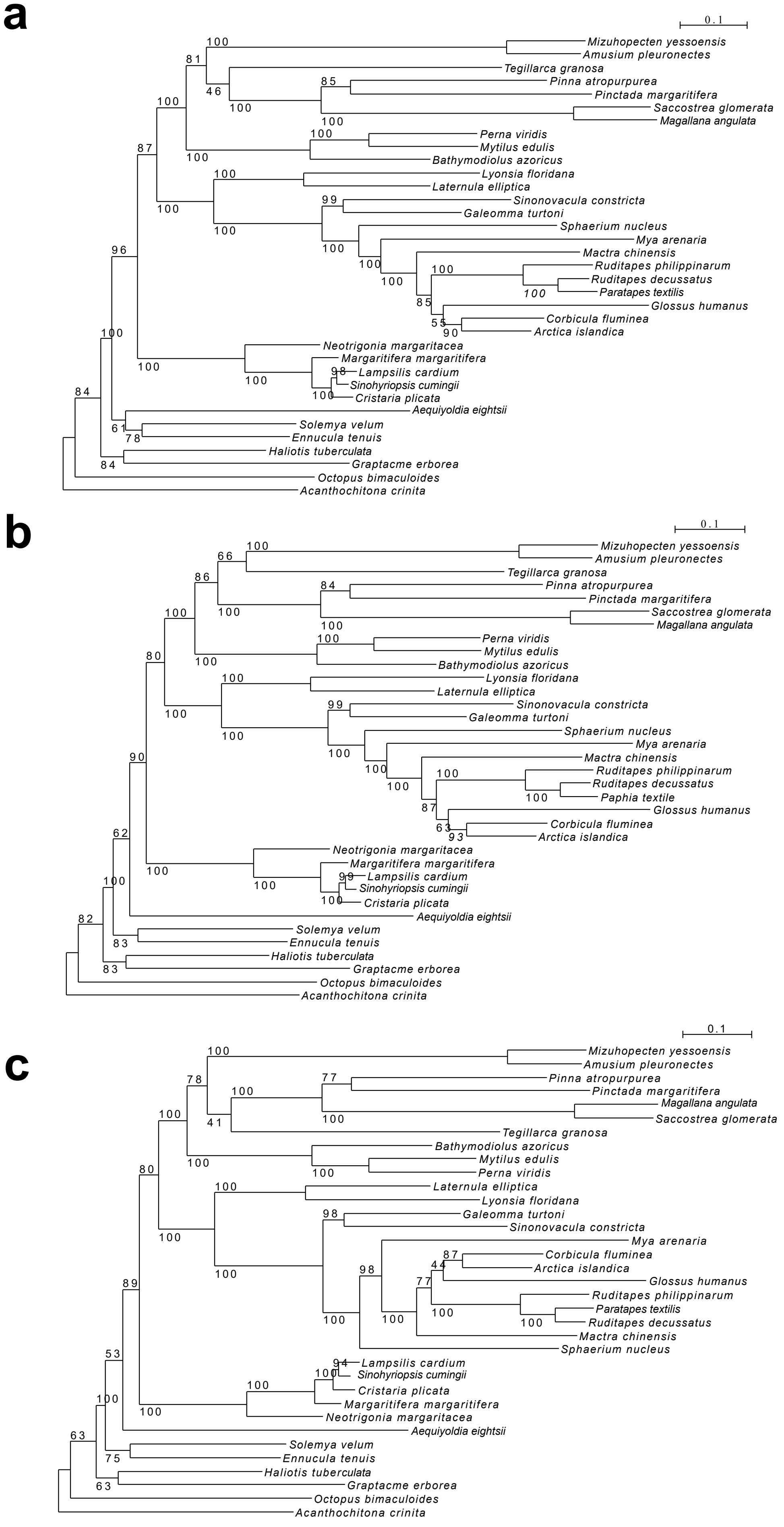

Figure S3. Trees inferred from the nuclear OXPHOS dataset. Each tree was obtained through a different phylogenetic pipeline (A) IQTREE partitioning the dataset, node support expressed as UFBoot value; (B) IQTREE with mixture model, node support expressed as UFBoot value; (C)RAxML, node support expressed as bootstrap value.

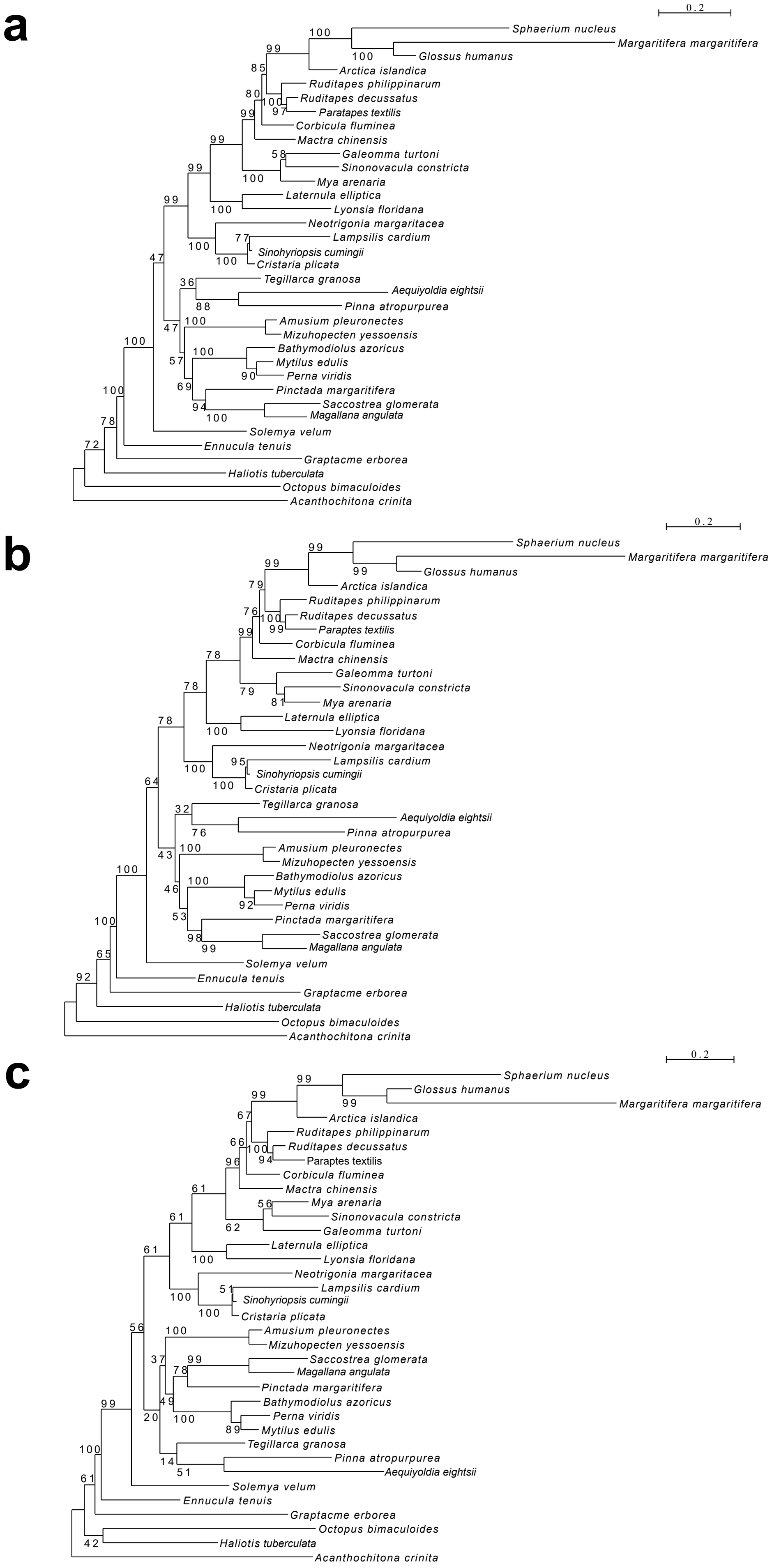

Figure S4. Trees inferred from the sncRNAs dataset. Each tree was obtained through a different phylogenetic pipeline (A) IQTREE partitioning the dataset, node support expressed as UFBoot value; (B) IQTREE with mixture model, node support expressed as UFBoot value; (C) RAxML, node support expressed as bootstrap value.

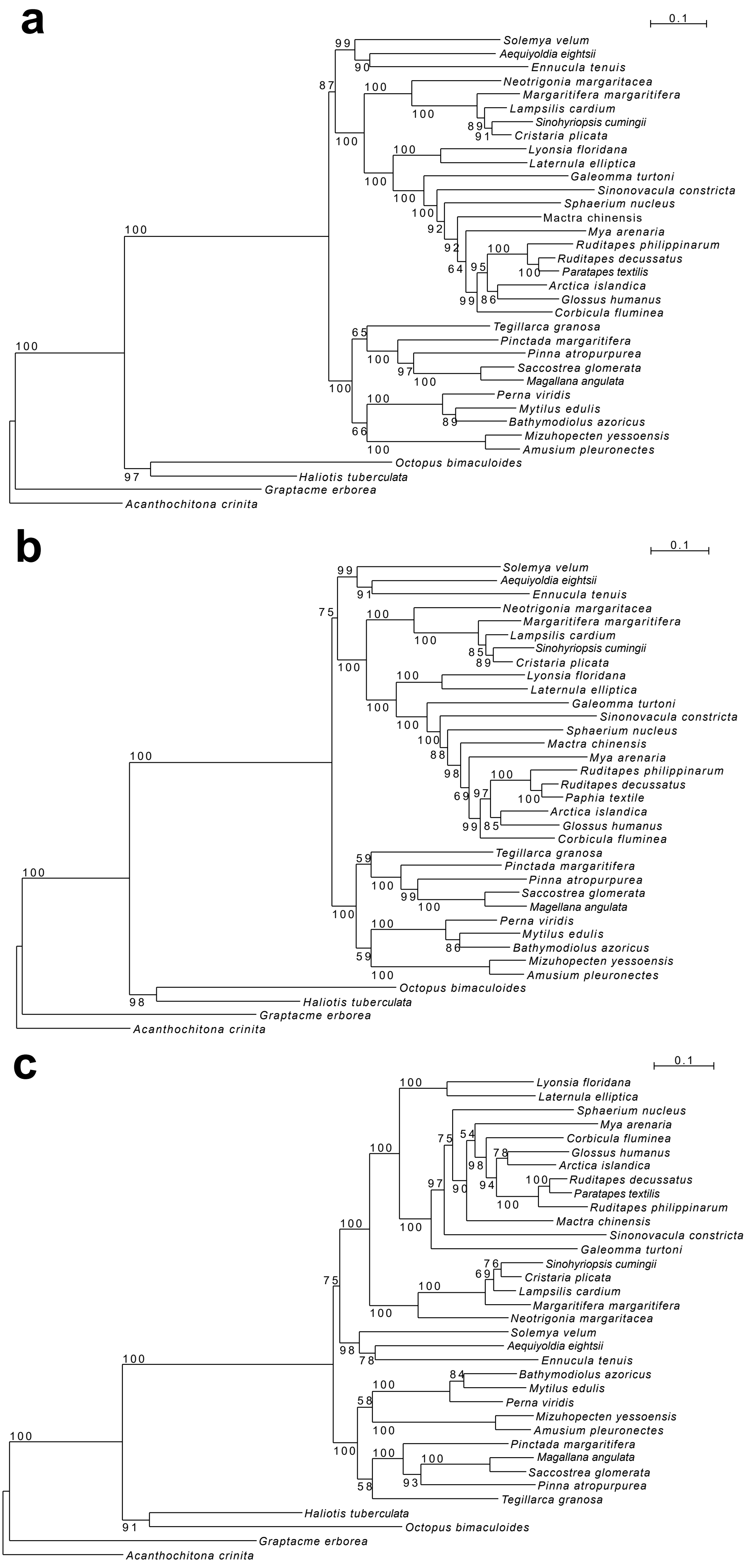

Figure S5. Trees inferred from the glycolysis dataset. Each tree was obtained through a different phylogenetic pipeline (A) IQTREE partitioning the dataset, node support expressed as UFBoot value; (B) IQTREE with mixture model, node support expressedas UFBoot value; (C) RAxML, node support expressed as bootstrap value.

Dataset	Sum ΔSLS	Average ΔSLS	%ΔSLSs > 0.5[c]	%ΔSLSs < −0.5[d]	mt-UFBoot	nuc-UFBoot	mt-gCF	nuc-gCF	mt-sCF	nuc-sCF
sncRNAs	−308.6	−0.079	7.76%	11.24%	0	99	0	27.8	28.9	46.8
glycolysis	−338.8	−0.021	0.69%	2.26%	0	100	5.0	32.5	31.1	41.2

Table S3. ΔSLSs, UFBoots and CFs calculated on the sncRNAs and glycolysis datasets for the mt and nuc-topology.

	*Acanthochitona avicula**	*Haliotis tuberculata*	*Octopus bimaculoides*	*Graptacme eborea*	*Solemya velum*
reference genome	NC_047426	NC_013708	NC_029723	NC_006162	NC_017612
atp6	red	blue	blue	blue	red
atp8	red	blue	blue	red	red
co1	red	blue	blue	blue	blue
co2	red	blue	blue	red	blue
co3	red	blue	blue	red	blue
cytb	blue	red	red	blue	red
nadh1	blue	red	red	red	red
nadh2	red	blue	blue	blue	blue
nadh3	red	blue	blue	blue	blue
nadh4	blue	red	red	red	blue
nadh4l	blue	red	red	red	blue
nadh5	blue	red	red	red	blue
nadh6	blue	red	red	red	red

Table S4. Mitochondrial genes localization in outgroups and Protobranchia OTUs. For each OTU it is reported the genes that are located on the G+T rich strand (in red) and the genes on the G+T poor strand (in blue) according to the reference mitochondrial genomes. Since no mitochondrial genome of *A. crinita* is available on NCBI, in this table we used the *A. avicula* mitochondrial reference genome. *Yoldia eightsii* and *Ennucula tenuis* are excluded from the table since no mitochondrial genome for these species or congeneric species has been annotated yet.

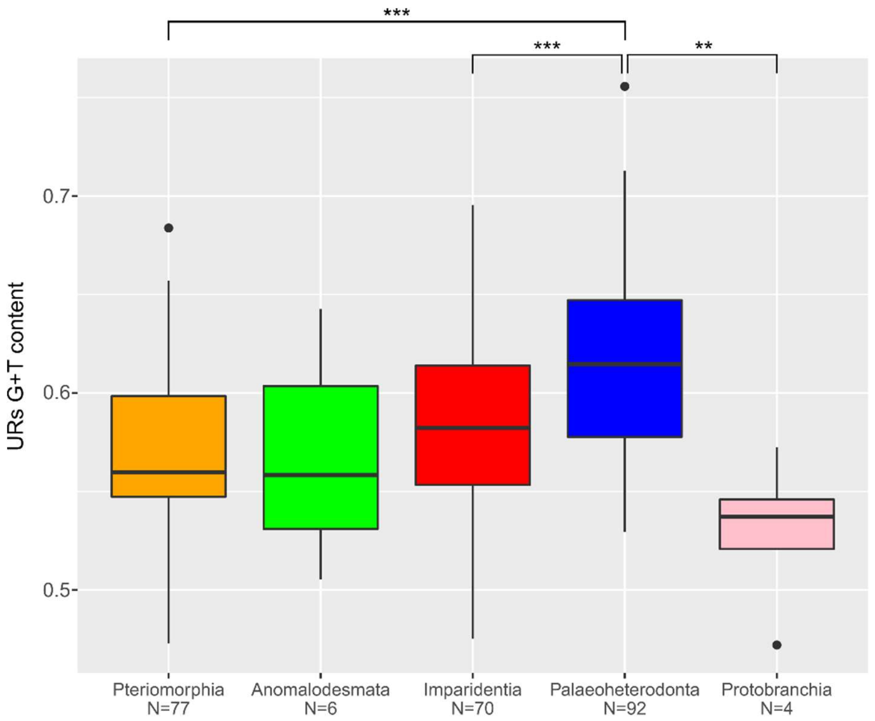

Figure S6. Boxplot comparing the mitochondrial unassigned regions G + T content between the five main Bivalvia clades. **, 0.01> p-value ≥0.001 and ***, p-value < 0.001. The significance of the comparisons was calculated through the Kruskal and Wallis test, followed by the Dunn's test with Bonferroni's correction The number of entries for each group is reported right below the clade label.

markers	G+T content	AT skew	GC skew
atp6	0.67085	−0.365079365	0.286384977
atp8			
co1	0.62756	−0.263736264	0.238596491
co2	0.60705	−0.174193548	0.282051282
co3	0.65468	−0.317431193	0.294117647
cytb	0.64876	−0.298636927	0.295081967
nadh1	0.65680	−0.31629393	0.307692308
nadh2	0.67647	−0.347945205	0.365517241
nadh3	0.73446	−0.477911647	0.447619048
nadh4	0.65303	−0.216216216	0.295566502
naadh4l	0.63836	−0.310722101	0.416666667
nadh5	0.65901	−0.322357019	0.308823529
nadh6	0.68659	−0.328165375	0.478787879

Table S5. *Yoldia eightsii* markers nucleotide composition. The table reports the G+T content, the AT skew and the GC skew calculated on the *Y. eightsii* mitochondrial markers. atp8 gene is missing.

Accession number	Species	Marker	Source database
O74957	*Homo sapiens*	*ago-01*	SwissProt
Q8CJG1	*Mus musculus*	*ago-01*	SwissProt
Q27IR0	*Drosophila melanogaster*	*ago-01*	SwissProt
K4I6K9	*Danio rerio*	*ago-01*	SwissProt
F1MXX2	*Bos taurus*	*ago-01*	SwissProt
Q6QME8	*Bos taurus*	*ago-02*	SwissProt
Q9VUQ5	*Drosophila melanogaster*	*ago-02*	SwissProt
Q9UKV8	*Homo sapiens*	*ago-02*	SwissProt
Q8CJG0	*Mus musculus*	*ago-02*	SwissProt
K4I874	*Danio rerio*	*ago-02*	SwissProt
A0A0U1RML6	*Caenorhabditis elegans*	*ago-02*	SwissProt
Q9H9G7	*Homo sapiens*	*ago-03*	SwissProt
A9ZSZ2	*Bombyx mori*	*ago-03*	SwissProt
Q7PLK0	*Drosophila melanogaster*	*ago-03*	SwissProt
Q8CJF9	*Mus musculus*	*ago-03*	SwissProt
F1MG44	*Bos taurus*	*ago-03*	SwissProt
Q9HCK5	*Homo sapiens*	*ago-04*	SwissProt
Q8CJF8	*Mus musculus*	*ago-04*	SwissProt
K4IAH1	*Danio rerio*	*ago-04*	SwissProt
Q6J5K9	*Drosophila melanogaster*	*armitage*	SwissProt
Q9HCE1	*Homo sapiens*	*armitage*	SwissProt
O76922	*Drosophila melanogaster*	*aubergine*	SwissProt
Q9V3G6	*Drosophila melanogaster*	*cnot10*	SwissProt
Q9H9A5	*Homo sapiens*	*cnot10*	SwissProt
A4IFB6	*Bos taurus*	*cnot10*	SwissProt
Q8BH15	*Mus musculus*	*cnot10*	SwissProt
KT694356.1	*Mytilus galloprovincialis*	*cnot10*	NCBI
Q08CL8	*Danio rerio*	*cnot10*	SwissProt
Q8WYQ5	*Homo sapiens*	*dgcr8*	SwissProt
Q8BH15	*Mus musculus*	*dgcr8*	SwissProt
A6QR44	*Bos taurus*	*dgcr8*	SwissProt
A2BEG3	*Danio rerio*	*dgcr8*	SwissProt
A0A210R4Q5	*Mizuhopecten yessoenensis*	*dgcr8*	SwissProt
KT447252.1	*Mytilus galloprovincialis*	*dgcr8*	NCBI
Q9VCU9	*Drosophila melanogaster*	*dicer*	SwissProt
Q6TV19	*Danio rerio*	*dicer*	SwissProt
Q6TUI4	*Bos taurus*	*dicer*	SwissProt
Q8R418	*Mus musculus*	*dicer*	SwissProt
A0A0B7C3X8	*Arion vulgaris*	*dicer*	SwissProt
A0A210QXA0	*Mizuhopecten yessoenensis*	*dicer*	SwissProt
KT447258.1	*Mytilus galloprovincialis*	*dicer*	NCBI
Q9NRR4	*Homo sapiens*	*drosha*	SwissProt
Q5HZJ0	*Mus musculus*	*drosha*	SwissProt
21349627.1	*Mizuhopecten yessoenensis*	*drosha*	NCBI
KT447251.1	*Mytilus galloprovincialis*	*drosha*	NCBI
Q8WWH4	*Homo sapiens*	*gasz*	SwissProt
Q8VD46	*Mus musculus*	*gasz*	SwissProt

Q8WMX6	*Pan troglodytes*	*gasz*	SwissProt
Q8WMX8	*Bos taurus*	*gasz*	SwissProt
13095931.1	*Biomphalaria glabrata*	*gasz*	NCBI
21362591.1	*Mizuhopecten yessoenensis*	*gasz*	NCBI
19927737.1	*Crassostrea gigas*	*gasz*	NCBI
4927861.1	*Bombyx mori*	*gasz*	NCBI
Q8SY33	*Drosophila melanogaster*	*gw182*	SwissProt
Q17740	*Caenorhabditis elegans*	*gw182*	SwissProt
Q9VNS0	*Drosophila melanogaster*	*maelstrom*	SwissProt
Q8BVN9	*Mus musculus*	*maelstrom*	SwissProt
Q96JY0	*Homo sapiens*	*maelstrom*	SwissProt
21362591.1	*Mizuhopecten yessoenensis*	*maelstrom*	NCBI
KT694366.1	*Mytilus galloprovincialis*	*maelstrom*	NCBI
Q504Q3	*Homo sapiens*	*pan2*	SwissProt
Q8BGF7	*Mus musculus*	*pan2*	SwissProt
21376239.1	*Mizuhopecten yessoenensis*	*pan2*	NCBI
KT694367.1	*Mytilus galloprovincialis*	*pan2*	NCBI
H3BKF3	*Mus musculus*	*pan3*	SwissProt
K1QVJ8	*Crassostrea gigas*	*pan3*	SwissProt
E7EXP6	*Danio rerio*	*pan3*	SwissProt
21364101.1	*Mizuhopecten yessoenensis*	*pan3*	NCBI
13064656.1	*Biomphalaria glabrata*	*pan3*	NCBI
Q8UVX0	*Danio rerio*	*piwia*	SwissProt
P22893	*Mus musculus*	*piwia*	SwissProt
Q9VKM1	*Drosophila melanogaster*	*piwia*	SwissProt
O61931	*Caenorhabditis elegans*	*piwia*	SwissProt
Q96J94	*Homo sapiens*	*piwia*	SwissProt
A8D8P8	*Bombyx mori*	*piwia*	SwissProt
K1QVJ8	*Crassostrea gigas*	*piwia*	SwissProt
A0A210PYP0	*Mytilus galloprovincialis*	*piwia*	SwissProt
E1B7E6	*Bos taurus*	*piwia*	SwissProt
D9I023	*Crepidula fornicata*	*piwia*	SwissProt
KT447255.1	*Mytilus galloprovincialis*	*piwia*	NCBI
A0A2C9JYQ3	*Biomphalaria glabrata*	*piwia*	SwissProt
A0A0P0M0F9	*Azumapecten ferreri*	*piwia*	SwissProt
Q8CDG1	*Mus musculus*	*piwib*	SwissProt
A2CEI6	*Danio rerio*	*piwib*	SwissProt
Q8TC59	*Homo sapiens*	*piwib*	SwissProt
A0A210Q0Y2	*Mytilus galloprovincialis*	*piwib*	SwissProt
K1QKA9	*Crassostrea gigas*	*piwib*	SwissProt
KT447256.1	*Mytilus galloprovincialis*	*piwib*	NCBI
O14744	*Homo sapiens*	*prmt5*	SwissProt
P46580	*Caenorhabditis elegans*	*prmt5*	SwissProt
Q9U6Y9	*Drosophila melanogaster*	*prmt5*	SwissProt
Q8CIG8	*Mus musculus*	*prmt5*	SwissProt
B0R026	*Danio rerio*	*prmt5*	SwissProt
A0A210PE51	*Mizuhopecten yessoenensis*	*prmt5*	SwissProt
11420415.1	*Crassostrea gigas*	*prmt5*	NCBI
13084844.1	*Biomphalaria glabrata*	*prmt5*	NCBI

P62826	*Homo sapiens*	*ran*	SwissProt
P62827	*Mus musculus*	*ran*	SwissProt
Q9VZ23	*Drosophila melanogaster*	*ran*	SwissProt
11426860.1	*Crassostrea gigas*	*ran*	NCBI
13094746.1	*Biomphalaria glabrata*	*ran*	NCBI
KT447254.1	*Mytilus galloprovincialis*	*ran*	NCBI
Q9W1I9	*Drosophila melanogaster*	*shutdown*	SwissProt
21353947.1	*Mizuhopecten yessoenensis*	*shutdown*	NCBI
13077159.1	*Biomphalaria glabrata*	*shutdown*	NCBI
Q7SXR1	*Danio rerio*	*tarbp2*	SwissProt
Q15633	*Homo sapiens*	*tarbp2*	SwissProt
Q0IIG6	*Bos taurus*	*tarbp2*	SwissProt
21375211.1	*Mizuhopecten yessoenensis*	*tarbp2*	NCBI
KT447253.1	*Mytilus galloprovincialis*	*tarbp2*	NCBI
P22893	*Mus musculus*	*tudor*	SwissProt
Q58EK5	*Danio rerio*	*tudor*	SwissProt
Q9BXT4	*Homo sapiens*	*tudor*	SwissProt
H9JD76	*Bombyx mori*	*tudor*	SwissProt
W5VJD8	*Biomphalaria glabrata*	*tudor*	SwissProt
K1R3H4	*Crassostrea gigas*	*tudor*	SwissProt
A0A210PGL2	*Mytilus galloprovincialis*	*tudor*	SwissProt
A0A126Q9D5	*Mytilus galloprovincialis*	*tudor*	SwissProt
Q8T0F1	*Drosophila melanogaster*	*tudor*	SwissProt
Q9NQI0	*Homo sapiens*	*vasa*	SwissProt
11437246.1	*Crassostrea gigas*	*vasa*	NCBI
21370695.1	*Mizuhopecten yessoenensis*	*vasa*	NCBI
Q9VKD7	*Drosophila melanogaster*	*zuc*	SwissProt
11428483.1	*Crassostrea gigas*	*zuc*	NCBI
13063678.1	*Biomphalaria glabrata*	*zuc*	NCBI
21352816.1	*Mizuhopecten yessoenensis*	*zuc*	NCBI

Table S6. List of entries included in the user-defined database for the annotation of transcripts related to the biogenesis of sncRNAs.

Dataset	Marker	KEGG ID	Orthologous groups
nuclear OXPHOS	*atpef1A*	K02132	1
	atpef1B	K02133	1
	atpef1G	K02136	1
	aptef1D	K02134	1
	atpef1E	K02135	1
	atpef0B	K02127	1
	atpef0C	K02128	1
	atpef0D	K02138	1
	atpef0E	K02129	1
	atpef0F	K02130	1

atpef0F6	K02131	1
atpef0fFO	K02137	1
atpefG	K02140	1
cox4	K02263	1
cox5a	K02264	1
cox5b	K02265	1
cox6a	K02266	1
cox6b	K02267	1
cox7a	K02270	1
cox7c	K02272	1
cox10	K02257	1
cox11	K02258	1
cox15	K02259	1
cox17	K02260	1
ndufa2	K03946	1
ndufa4	K03948	1
ndufa5	K03949	1
ndufa6	K03950	1
ndufa7	K03951	1
ndufa8	K03952	1
ndufa9	K03953	1
ndufa10	K03954	1
nudfa12	K11352	1
ndufa13	K11353	1
ndufb1	K03957	1
ndufb2	K03958	1
ndufb3	K03959	1
ndufb4	K03960	1
ndufb5	K03961	1
ndufb6	K03962	1
ndufb7	K03963	1
ndufb8	K03964	1
ndufb9	K03965	1
ndufb10	K03966	1
ndufb11	K11351	1
ndufs1	K03934	1
ndufs2	K03935	1
ndufs3	K03936	1
ndufs4	K03937	1
ndufs5	K03938	1
ndufs6	K03939	1
ndufs7	K03940	1
ndufs8	K03941	1
ndufv1	K03942	1
ndufv2	K03943	1
ndufv3	K03944	1
qcr2	K00415	1

	qcr6	K00416	1
	qcr7	K00417	1
	qcr8	K00418	1
	qcr9	K00419	1
	cyc1	K00413	1
	uqcrfs1	K00411	1
	sdha	K00234	1
	sdhb	K00235	1
	sdhc	K00236	1
	sdhd	K00237	1
Mitochondrial OXPHOS	*atp6*	K02126	1
	atp8	K02125	1
	co1	K02256	1
	co2	K02261	1
	co3	K02262	1
	cytb	K00412	1
	nadh1	K03878	1
	nadh2	K03879	1
	nadh3	K03880	1
	nadh4	K03881	1
	nadh4L	K03882	1
	nadh5	K03883	1
	nadh6	K03884	1
sncRNA	*ago-01*	K11593	1
	ago-02	K11593	1
	armitage	K18432	1
	cnot10	K12607	1
	dgcr8	K18418	1
	dicer	K11592	1
	drosha	K03685	1
	gasz	K18410	1
	gw182	K18412	1
	maelstrom	K18411	1
	pan2	K12571	1
	pan3	K12572	1
	piwiA	K02156	1
	piwiB	K02156	1
	prmt5	K02516	1
	ran	K07936	1
	sht	K09571	1
	tarbp2	K18420	1
	tudor	K18407	1
	vasa	K13982	1
	zucchini	K16862	1
partial-glyco	*adh*	K00001	1

	ldh	K00016	1
	adhC	K00121	1
	pdhA	K00161	1
	pdhB	K00162	1
	pdhD	K00382	1
	pyk	K00850	1
	pgk	K00927	1
	g6pc	K01084	1
	E5.1.3.15	K01792	1
	tpiA	K01803	1
	pgi	K01810	1
	gpmA	K01834	1
	pgm	K01835	1
	fbp	*K03841*	1
	aldh741	K14085	1
	pgm2	K15779	1
total-glyco	*akr1a1*	K00002	3
	aldh	K00128	3
	aldh3	K00129	1
	aldh9a1	K00149	2
	pdhc	K00627	2
	hk	K00844	2
	pckA	K01596	3
	aldo	K01623	2
	eno	K01689	2
	galM	K01785	2
	acs	K01895	2
	minnp1	K03103	2
	adpgk	K08074	2
	adph	K13953	1

Table S7. List of entries included in the user-defined database for the annotation of transcripts related to the biogenesis of sncRNAs. For each markers is reported the number of clear monophyletic group detected, which are more than 1 only in the total-glyco dataset.
